# What makes a “successful” or “unsuccessful” discharge letter? Hospital clinician and General Practitioner assessments of the quality of discharge letters

**DOI:** 10.1186/s12913-021-06345-z

**Published:** 2021-04-15

**Authors:** Katharine Weetman, Rachel Spencer, Jeremy Dale, Emma Scott, Stephanie Schnurr

**Affiliations:** 1 Unit of Academic Primary Care, Warwick Medical School, University of Warwick, Coventry, England CV4 7AL UK; 2 Applied Linguistics, University of Warwick, Coventry, UK

**Keywords:** Discharge summaries, Discharge communication, Patient safety, Continuity of care, Discharge letters, Doctor and patient communication, Hospital discharge, Inter-professional communication

## Abstract

**Background:**

Sharing information about hospital care with primary care in the form of a discharge summary is essential to patient safety. In the United Kingdom, although discharge summary targets on timeliness have been achieved, the quality of discharge summaries’ content remains variable.

**Methods:**

Mixed methods study in West Midlands, England with three parts: 1. General Practitioners (GPs) sampling discharge summaries they assessed to be “successful” or “unsuccessful” exemplars, 2. GPs commenting on the reasons for their letter assessment, and 3. surveying the hospital clinicians who wrote the sampled letters for their views. Letters were examined using content analysis; we coded 15 features (e.g. “diagnosis”, “GP plan”) based on relevant guidelines and standards. Free text comments were analysed using corpus linguistics, and survey data were analysed using descriptive statistics.

**Results:**

Fifty-three GPs participated in selecting discharge letters; 46 clinicians responded to the hospital survey. There were statistically significant differences between “successful” and “unsuccessful” inpatient letters (*n* = 375) in relation to inclusion of the following elements: reason for admission (99.1% vs 86.5%); diagnosis (97.4% vs 74.5%), medication changes (61.5% vs 48.9%); reasons for medication changes (32.1% vs 18.4%); hospital plan/actions (70.5% vs 50.4%); GP plan (69.7% vs 53.2%); information to patient (38.5% vs 24.8%); tests/procedures performed (97.0% vs 74.5%), and test/examination results (96.2% vs 77.3%). Unexplained acronyms and jargon were identified in the majority of the sample (≥70% of letters). Analysis of GP comments highlighted that the overall clarity of discharge letters is important for effective and safe care transitions and that they should be relevant, concise, and comprehensible. Hospital clinicians identified several barriers to producing “successful” letters, including: juniors writing letters, time limitations, writing letters retrospectively from patient notes, and template restrictions.

**Conclusions:**

The failure to uniformly implement national discharge letter guidance into practice is continuing to contribute to unsuccessful communication between hospital and general practice. While the study highlighted barriers to producing high quality discharge summaries which may be addressed through training and organisational initiatives, it also indicates a need for ongoing audit to ensure the quality of letters and so reduce patient risk at the point of hospital discharge.

**Supplementary Information:**

The online version contains supplementary material available at 10.1186/s12913-021-06345-z.

## Background

Discharge from hospital is a high-risk healthcare event; risk of harm can originate in the secondary care setting [1] or the primary care setting [2, 3], This is particularly well evidenced in relation to medications errors following discharge [4]. Sharing accurate, relevant information about the care received in hospital with primary care in the form of a discharge summary is essential to patient safety. This process inherently involves inter-professional communication which has been previously suggested as an area that requires improvement [5, 6]. Poor discharge communication is an important cause of adverse events in medical defence organisation data [7] and incident reports from primary care [8–10]. Two inter-related elements are at play: speed of information transfer and quality of information transferred. International evidence suggests that discharge letters are unsatisfactory for a number of reasons including: incomplete and insufficient information [11–13], unclear follow up plans [12, 14, 15], letter inaccuracies [16], delayed letter delivery [12, 17, 18], inadequate medication information [19, 20], lack of patient-centredness (e.g. letter dense with jargon) [12, 21, 22] and general communication gaps leading to adverse events such as patient readmissions [23, 24].

In the United Kingdom (UK), hospitals have been required to use electronic discharge summaries since 2015, and the benefits of such summaries are well established [25]. Hospitals are now required to produce summaries within 24 h [26]. Our previous study [2], conducted in 2016, in general practices in three different areas of the UK, shows that summaries arrive and are uploaded to General Practitioner (GP) systems in a time efficient manner (median of two days from discharge). Although targets on timeliness appear to have been achieved, the quality of discharge summaries’ content remains less certain and there is now a need to focus on this as a route to improved patient safety during care transitions.

The Discharge Communication Study [27] (of which this research is a part) investigated ways of improving the content and processes surrounding discharge letters. Results relating to GP interviews contextualising elements of successful letters [28], and patient interviews (which investigate patient-provider communication) [29] have already been published elsewhere. In this paper we report clinicians’ opinions on the quality of discharge letters which are crucial to understanding how to improve inter-professional communication at the time of discharge. We investigated quality from the perspectives of ‘instigator’ (hospital) and ‘receiver’ (primary care) through surveys and discharge letter analysis; this is important given that GPs manage patient care when they are discharged back to the community, as is comparing these views with that of the hospital clinician who is responsible for managing patient care in hospital and writing the discharge letter. This paper describes from hospital clinician and GP perspectives what makes a “successful” or “unsuccessful” discharge letter. The research questions were:

1. What do hospital clinicians and GPs judge to be the features and key content-items of “successful” discharge letters?
2. What do hospital clinicians view as barriers to producing “successful” or high-quality discharge letters?

## Methods

### Recruitment and data collection

To explore what makes a “successful” or “unsuccessful” discharge letter, our mixed methods study gathered data across the West Midlands, England, from three sources: 1. discharge letters sampled by GPs as reflecting “successful” or “unsuccessful” exemplars, 2. comments by the GPs to explain their letter gradings, and 3. a hospital clinician survey.

GP recruitment and discharge letter sampling took place between August 2017 and April 2018. As stated in our published study protocol [27], *“the study aimed to recruit 30-50 GPs across 15 practices, with a target of 2-3 GPs per practice”* (p.5). Furthermore, the study protocol [27] describes that participating GPs were asked to select 14–24 recent (< 3 weeks) discharge letters which they assessed to be “successful” or “unsuccessful” exemplars. They were asked to select these from letters relating to adult (18+ years) patients discharged from an NHS hospital in Warwickshire, Coventry, Rugby, Herefordshire or Worcestershire following an episode of inpatient or outpatient care, excluding discharge letters from mental health services or related to patients who lacked capacity to consent [27] (for further details and full justification of the inclusion and exclusion criteria, see our study protocol [27]). GP letter selection identified potential participants for all subsequent phases relating to the Discharge Communication Study [27], including patients eligible for interview (these results are published separately [29]).

There were no set criteria for letter gradings of “successful” and “unsuccessful” as it was purposefully intended to reflect each participating GP’s individual interpretation and experiences of whether the letter communicated necessary, important discharge information [27]. The GPs were asked to complete a selection template (additional file 1) to record their letter gradings and comments on their reasons for the letter categorisations [27]. As stated in our protocol [27], *“GP practice staff redacted the letters of patient identifiable information”* (p.5). After redaction [27], letters were transferred to the research team for analysis of the GP comments as well as assessment of the letters themselves through content analysis (see analysis section below).

Hospital clinicians who were identified as having written a letter included in those sampled by the GPs were invited to take part in a survey (see additional file 2). This asked them to assess the letter they wrote in relation to different content items (e.g. diagnosis information) and for different audiences (e.g. GP or patient). The survey was open from May to September 2018. Survey packs were sent by post or distributed by internal mail within the hospital [27]; packs contained an invitation letter explaining why they had been selected, a participant information sheet, the survey, and a redacted copy of the sampled discharge letter [27]. Where a clinician had been included in the sample on more than one occasion, they only received a survey pack related to one letter that had been included in the sample; letters were prioritised by whether the patient and GP had taken part in interviews (see protocol [27] for further details of this letter and case matching process). The invitation letter stated that completing and returning a survey indicated consent. The survey asked respondents to select numerical ratings on a “semantic differential” [30, 31] scale from 1 to 9, with 1 being ‘low’ and 9 being ‘high’; each end of the scale featured bipolar evaluative adjectival phrases e.g. “uninformative/ informative”. Adjectives were selected for measuring varying dimensions of beliefs toward the letter quality. To encourage participation the survey was kept relatively short; 14 of the questions were closed with one open question at the end of the survey (see additional file 2).

### Analysis

#### Discharge letter content analysis

Discharge letters were analysed using *content analysis* [32–34] which is a systematic approach to qualitative textual data utilising coding techniques in order to deduce findings [32, 34]. Our content analysis aimed to examine possible associations between the content of letters and GP quality gradings to address the research questions. The alternative (H1) and null hypothesis (H0) are below:

H1: There is an association between [letter content features] and GP letter gradings.

H0: There is no association between [letter content features] and GP letter gradings.

Discharge letter standards [35–38] were used to structure assessment of the quality of the letter sample in terms of content components (e.g. diagnosis). Guidelines released after letter sampling [39] were not used due to unfairness in evaluating letters against guidelines not yet published at the time of letter production (e.g. 2019 eDischarge standards [40]). Content items from guidelines were selected and synthesised with respect to the research questions. Coding categories (e.g. procedures performed) were primarily based around core clinical elements (e.g. investigation results) and informational needs of patients (e.g. jargon explained) and GPs (e.g. GP plan). Letters were coded as to whether they contained content components and thus adhered to guidelines and standards.

The initial coding system for content analysis had 20 categories; this was piloted with a 5% sample coded independently by KW and JD to assess coding reliability. Inter-coder agreement was generally satisfactory (Table 1) (k > 0.8) [41]; the median across scores was 0.94. Two categories had unsatisfactory agreement: “reasons for medication” (k = .791) and “medical jargon” (k = .584). Discussion revealed differences in coding of implicit and explicit reasons for medication and whether jargon was explained from the lay or expert clinical perspective. These issues were resolved with minor revisions to the categorisation system (merging two categories, and removal of four categories due to category coding overlap). The first author coded the remaining 95% of the sample; difficult cases were discussed with the team. The final content analysis framework contained 15 categories (see additional file 3 for full list) which included:

1. Discharging physician details (name and role of discharging physician)
2. Clinical summary elements (reason for admission, diagnosis, procedures/investigations performed and results)
3. Any pending hospital plans or actions such as follow up in outpatient clinic
4. Medication information (name and dosing, details of changes and reasons)
5. GP plan (follow up, actions and management recommendations)
6. Details of patient wishes/concerns as well as information provided to patient
7. Elements pertaining to letter content style (e.g. whether acronyms explained).

**Table 1:** Results of inter-coder agreement for pilot content coding

Categorisation heading for coding	Kappa agreement measure
Discharging speciality/department	.881 (*p* < 0.001)
Discharging consultant	.940 (*p* < 0.001)
Reason for admission	.940 (*p* < 0.001)
Diagnosis	.940 (*p* < 0.001)
Procedures and investigations performed	.940 (*p* < 0.001)
Clinical summary	.970 (*p* < 0.001)
Investigation results	.881 (*p* < 0.001)
Examination findings	.851 (*p* < 0.001)
Medication name(s)	.970 (*p* < 0.001)
Medication dose and frequency	.970 (*p* < 0.001)
Reasons for medication	.791 (*p* < 0.001)
Medication recommendations	.911 (*p* < 0.001)
Medication changes	.910 (*p* < 0.001)
Reasons for medication changes	.881 (*p* < 0.001)
Investigations/procedures/appointments requested of where results are pending	.940 (*p* < 0.001)
Patient’s and carer’s concerns, expectations and wishes	1.00 (*p* < 0.001)
Information and advice given to patient	.940 (*p* < 0.001)
Plan and requested actions/follow up	.911 (*p* < 0.001)
Acronyms (unexplained)	.940 (*p* < 0.001)
Medical jargon (unexplained)	.584 (*p* < 0.001)

Following piloting it was decided that the content coding should focus solely on the sampled inpatient letters. This was intended to increase the homogeneity of the sample.

“Successful” and “unsuccessful” coding results for the letters were quantitatively compared using Chi-square [42] (*p* < 0.05) in order to test hypotheses that there are content feature differences between GP-assessed “successful” and “unsuccessful” letters [27]. As the cross-tabulations for feature coding were 2 × 2, Yates’ correction was used [43]. We also compared Yates and Fishers corrections for all chi-square results; there were no significant differences.

#### Discharge letter comment analysis

Corpus linguistics [44, 45] is the study of collections of texts or *corpora* [46] and was used to analyse the GP comments relating to their quality assessment of sampled discharge letters. Corpus linguistics focuses on analysing patterns [47] and has previously successfully been applied to comment data within healthcare [48, 49]. Frequency and relative frequency results were compared between the “successful” and “unsuccessful” comment sub-corpora in order to expound patterns of relevance to the research questions. Concordance software *Antconc* [50] was used for compiling the corpus and generating outputs.

#### Hospital clinician survey analysis

Survey results were analysed using descriptive statistics in Statistical Package for the Social Sciences (*SPSS)*. Tests were nonparametric [51, 52] to avoid making distributional assumptions about the survey responses [43]. Hospital clinician free text comments were narratively overviewed using a rudimentary thematic analysis [53, 54] by KW whereby comments were read, re-read, and then annotated for ideas and concepts relating to the research questions; these were then reviewed and iteratively refined through discussion with other members of the research team (ES, JD, SS). During this process, “themes” began to emerge which were iteratively refined.

Agreement analyses using descriptive percentages were also undertaken to compare the GP and hospital letter ratings. Kappa measures [55, 56] were run to look at the proportion of agreement between the GP and hospital respondents. GP gradings were binary (successful/unsuccessful) while HP gradings were on an interval scale 1–9. Consequently, as the kappa test requires and assumes two categorical variables with equal categories [43], HP gradings were recoded into unsuccessful (assessment scores 1–5) and successful (scores 6–9).

## Results

### Recruitment and data collection

**Fig. 1:**
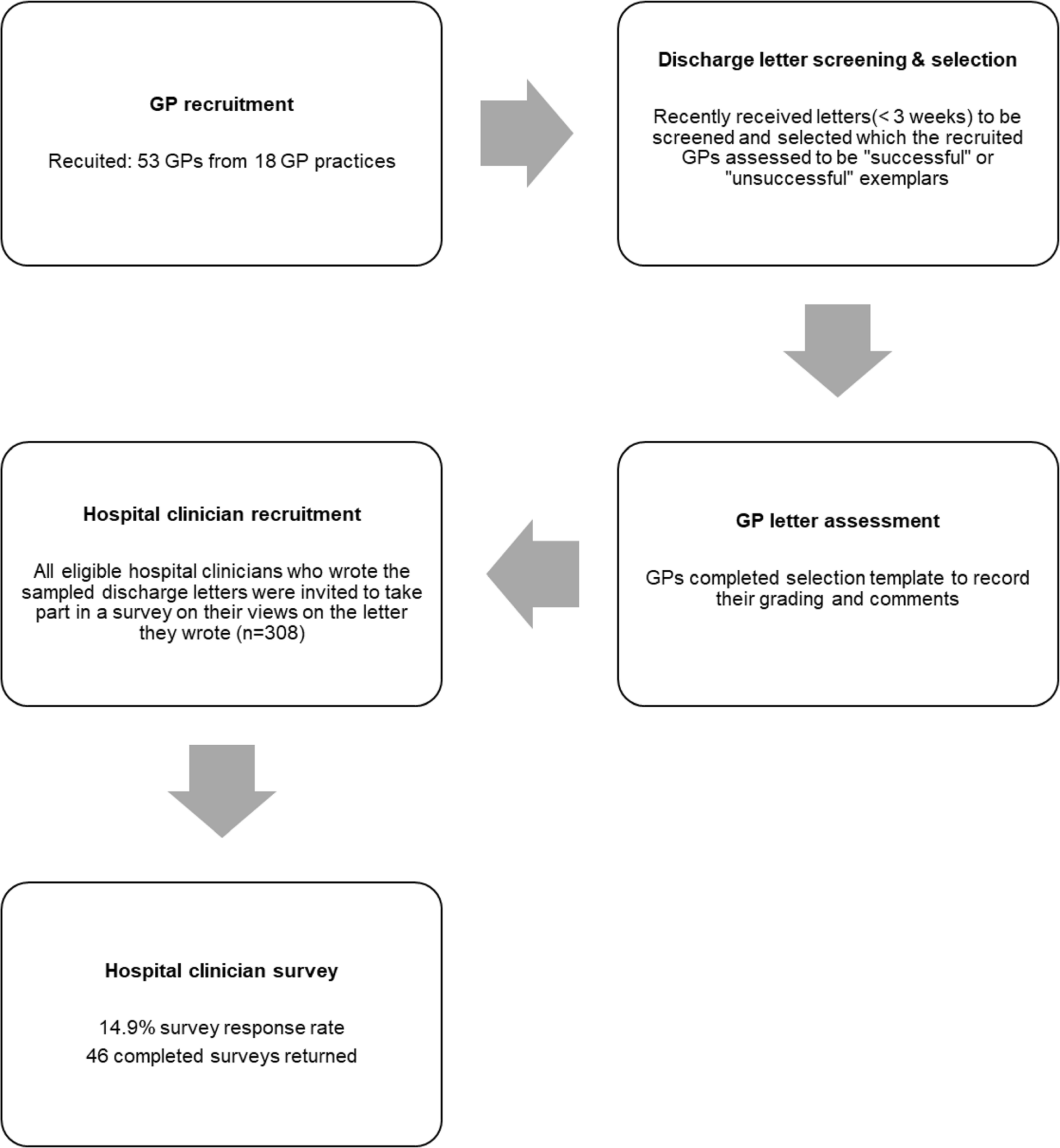
Data collection and recruitment targets and results

The study recruited 53 GPs across 18 practices with a median of 3 GPs per practice; range 1–8 GPs (see Fig. 1 for flowchart of data collection and recruitment). There was a median selection of 10 letters per GP, leading to a total sample of 489 discharge letters. 375 (76.7%) of these were inpatient discharge summaries; 234 (62.4%) were graded “successful” and 141 (37.6%) “unsuccessful”. The letters mainly related to patient care from four hospital trusts, and spanned 33 specialties with over 300 different reasons for care. A summary of GP practice and letter characteristics is in Table 2.

**Table 2:** Summary of inpatient discharge letter sample and GP practice characteristics

Demographics and characteristics	Inpatient discharge letter sample (***n*** = 375)	
	**Group**	**Frequency (%)**
GP letter grading	Successful	234 (62.4%)
	Unsuccessful	141 (37.6%)
	Total	375 (100%)
GP practice sizes	Small (< 5000 patients)	1 (5.6%)
	Medium (5–10,000 patients)	11 (61.1%)
	Large (10,000+ patients)	6 (33.3%)
Discharge speciality	missing	88 (23.5%)
	Accident & Emergency	10 (2.7%)
	Acute Medicine	7 (1.9%)
	Ambulatory care	2 (0.5%)
	Acute Medical Unit	2 (0.5%)
	Breast Surgery	2 (0.5%)
	Cardiology	22 (5.9%)
	Cardiothoracic Surgery	7 (1.9%)
	Clinical Haematology	6 (1.6%)
	Colorectal Surgery	8 (2.1%)
	Diabetic Medicine	5 (1.3%)
	Endocrinology	6 (1.6%)
	Ear Nose and Throat	5 (1.3%)
	Fetal Medicine	1 (0.3%)
	Gastroenterology	4 (1.1%)
	Gastrointestinal Surgery	6 (1.6%)
	General Medicine	32 (8.5%)
	General Surgery	26 (6.9%)
	Geriatric Medicine	15 (4.0%)
	Infectious diseases	3 (0.8%)
	Maxillo-facial surgery	2 (0.5%)
	Nephrology	10 (2.7%)
	Neurology	2 (0.5%)
	Neurosurgery	3 (0.8%)
	Obstetrics & Gynaecology	22 (5.9%)
	Oncology	3 (0.8%)
	Ophthalmology	1 (0.3%)
	Pancreatic Surgery	1 (0.3%)
	Plastic Surgery	1 (0.3%)
	Respiratory Medicine	21 (5.6%)
	Stroke Medicine	7 (1.9%)
	Trauma & Orthopaedics	16 (4.3%)
	Urology	21 (5.6%)
	Vascular Surgery	8 (2.1%)
	Total	375 (100.0%)
Letter format	combination	14 (3.7%)
	handwritten	5 (1.3%)
	typed	356 (94.9%)
	Total	375 (100.0%)
Sex of patient	missing	181 (48.3%)
	Female	94 (25.1%)
	Male	100 (26.7%)
	Total	375 (100.0%)
Age of patient	Range	20–96
	Median	64
	IQR	38, 77
Role of signing physician	Nurse/ACP	24 (6.4%)
	Junior Doctor	177 (47.2%)
	Speciality Trainee/ Core Trainee/ Registrar	80 (21.3%)
	Consultant	14 (3.7%)
	Other or unclear	80 (21.3%)
	Total	375 (100.0%)

Hospital clinicians eligible to participate in the survey were defined as the authors of the discharge letters. There were some letters with no named author or author name illegible, or where the author had left the trust. As a result, 308 doctors, nurses and allied health professionals were invited to take part in the survey, leading to responses from 46 hospital clinicians (response rate = 14.9%, see Fig. 1). A summary of responder characteristics is found in Table 3. Response rates ranged between hospital sites (5.4–33.3%). Variation was seen in age (24–60 years) and experience of respondents (qualifying year 1982–2017) although a large portion of surveys were completed by consultants (*n* = 19, 41.3%). Gender was self-described and in 74% of surveys, this information was optionally provided (15 female - 44.1%, 19 male – 55.9%). Interesting, but not statistically significant, trends were observed: junior doctors (*n* = 9) produced a low proportion of successful letters (22.2%), nurses/Advanced Clinical Practitioners (ACPs) (*n* = 10) produced a high proportion of successful letters (80.0%), and Acute Medicine (*n* = 5) and Cardiology (*n* = 5) had high weightings of successful letters (80.0 and 100.0% respectively).

**Table 3:** Summary of hospital respondents and relating discharge letter characteristics

Demographics and characteristics	Sample results	
	Group	Frequency (%)
GP letter grading	Successful	25 (54.3%)
	Unsuccessful	21 (45.7%)
	Total	46 (100%)
Discharge speciality	None specified	4 (8.7%)
	Accident & Emergency	4 (8.7%)
	Acute Medicine	5 (10.9%)
	Ambulatory care	1 (2.2%)
	Cardiology	5 (10.9%)
	Cardiothoracic Surgery	2 (4.3%)
	Care of the Elderly	2 (4.3%)
	Colorectal Surgery	1 (2.2%)
	Day Surgery	2 (4.3%)
	Ear Nose and Throat	3 (6.5%)
	General Medicine	2 (4.3%)
	General Surgery	3 (6.5%)
	Gynaecology	2 (4.3%)
	Pain service	1 (2.2%)
	Plastics	1 (2.2%)
	Respiratory	1 (2.2%)
	Trauma & Orthopaedics	4 (8.7%)
	Urology	3 (6.5%)
	Total	46 (100.0%)
Gender of hospital respondent	Missing	12 (26.1%)
	Female	15 (32.6%)
	Male	19 (41.3%)
	Total	46 (100.0%)
Age of hospital respondent	Missing	12 (26.1%)
	21–30	9 (19.6%)
	31–40	10 (21.7%)
	41–50	9 (19.6%)
	51–60	6 (13.0%)
	Total	46 (100.0%)
Role of signing physician	Other	1 (2.2%)
	Nurse/ACP	10 (21.7%)
	Junior Doctor	9 (19.6%)
	Speciality Trainee/ Core Trainee/ Registrar	7 (15.2%)
	Consultant	19 (41.3%)
	Total	46 (100.0%)
Ethnicity of hospital respondent	Missing	14 (30.4%)
	African	2 (4.3%)
	Asian	4 (8.7%)
	British	5 (10.9%)
	White	9 (19.6%)
	White British	11 (23.9%)
	Irish	1 (2.2%)
	Total	46 (100.0%)
Training location of hospital respondent	Internationally	6 (13.0%)
	Nationally (UK)	40 (87.0%)
	Total	46 (100.0%)
Hospital respondent year qualified	1981–1990	7 (15.2%)
	1991–2000	12 (26.1%)
	2001–2010	12 (26.1%)
	2011–2017	15 (32.6%)
	Total	46 (100.0%)
Religion of hospital respondent	Missing	27 (58.7%)
	Atheist	3 (6.5%)
	Christian	11 (23.9%)
	Hindu	3 (6.5%)
	Islam	2 (4.3%)
	Total	46 (100.0%)

### Features of successful discharge letters

Content analysis revealed that no feature was present in all discharge letters (Table 4). Highly frequent (≥80% of letters) content components were: reason for admission (94.4%), diagnosis (88.8%), tests/procedures performed (88.5%), investigation results/examination findings (89.1%), discharging physician name and role (84.0%), medication names (82.4%), medication dose and frequency (82.1%). Somewhat frequent (51–80% of letters) content components were: GP plan (63.5%), hospital plan (62.9%), medication changes (56.8%). For the following features, there were statistically significant differences between GP-assessed “successful” and “unsuccessful” letters (see Table 5):

- Reason for admission (99.1% successful, 86.5% unsuccessful, *X*^2^ = 24.176, *p* < 0.001)
- Diagnosis (97.4% successful, 74.5% unsuccessful, *X*^2^ = 44.386, *p* < 0.001),
- Medication changes (61.5% successful, 48.9% unsuccessful, *X*^2^ = 5.193, *p* = 0.023)
- Reasons for medication changes (32.1% successful, 18.4% unsuccessful, *X*^2^ = 7.606, *p* = 0.006)
- Hospital plan/actions (70.5% successful, 50.4% unsuccessful, *X*^2^ = 14.475, *p* < 0.001)
- GP plan (69.7% successful, 53.2% unsuccessful, *X*^2^ = 9.591, *p* = 0.002)
- Information to patient (38.5% successful, 24.8% unsuccessful, *X*^2^ = 6.764, *p* = 0.009)
- Tests/procedures performed (97.0% successful, 74.5% unsuccessful, *X*^2^ = 41.841, *p* < 0.001)
- Test/examination results (96.2% successful, 77.3% unsuccessful, *X*^2^ = 30.194, *p* < 0.001).

**Table 4:** Descriptive statistics for inpatient discharge letters content coding (n = 375)

Content feature	Feature presence (yes)	Feature absence (no)
1. Discharging physician (name and role)	84.0%	16.0%
2. Reason for admission	94.4%	5.6%
3. Diagnosis	88.8%	11.2%
4. Tests/procedures performed	88.5%	11.5%
5. Investigation results/examination findings	89.1%	10.9%
6. Medication names	82.4%	17.6%
7. Medication dose and frequency	82.1%	17.9%
8. Medication changes	56.8%	43.2%
9. Reasons for medication changes	26.9%	73.1%
10. Hospital plan (pending actions e.g. outpatient appointment)	62.9%	37.1%
11. Patient concerns/wishes	8.3%	91.7%
12. Information to patient	33.3%	66.7%
13. GP plan (actions/management)	63.5%	36.5%
14. Acronyms (unexplained)	81.3%	18.7%
15. Medical jargon (unexplained)	76.5%	23.5%

**Table 5:** Summary of statistical findings for letter content features against GP gradings

Content feature (independent variable)	Present in successful letters	Present in unsuccessful letters	Test	Df.*	*n*	χ^2^ value	Effect size -phi coefficient	*P* value
Discharging physician (name & role)	86.8%	79.4%	χ^2^	1	375	2.984	−.097	0.084
Reason for admission	99.1%	86.5%	χ^2^	1	375	24.176	−.266	< 0.001
Diagnosis	97.4%	74.5%	χ^2^	1	375	44.386	−.353	< 0.001
Tests/procedures performed	97.0%	74.5%	χ^2^	1	375	41.841	−.343	< 0.001
Investigation results	96.2%	77.3%	χ^2^	1	375	30.194	−.293	< 0.001
Medication names	84.6%	78.7%	χ^2^	1	375	1.719	−.075	0.190
Medication dose & frequency	84.2%	78.7%	χ^2^	1	375	1.437	−.069	0.231
Medication changes	61.5%	48.9%	χ^2^	1	375	5.193	−.123	0.023
Reasons for changes	32.1%	18.4%	χ^2^	1	375	7.606	−.149	0.006
Hospital plan/actions	70.5%	50.4%	χ^2^	1	375	14.475	−.202	< 0.001
Patient concerns/wishes	8.1%	8.5%	χ^2^	1	375	.000	.007	1.000
Information to patient	38.5%	24.8%	χ^2^	1	375	6.764	−.140	0.009
GP plan & actions	69.7%	53.2%	χ^2^	1	375	9.591	−.166	0.002
Acronyms (unexplained)	78.2%	86.5%	χ^2^	1	375	3.482	.103	0.062
Medical jargon (unexplained)	71.8%	84.4%	χ^2^	1	375	7.095	.144	0.008

*Df. degrees of freedom

The GP comment corpus (*n* = 375) comprised 4804 words and was divided into two sub-corpora formed of 189 (50.4%) “successful” graded letter comments (2093 words) and 186 (49.6%) “unsuccessful” graded letter comments (2711 words). The 25 most frequent content words (excluding functional words e.g. to/of/and) are in Table 6. As the number of comments was felt to be a manageable number, a saturation point [48] was not required and all comments were examined. A summary of qualitative findings relating to GP comments is found in Table 7. Notably, the most frequent word in the “successful” comment sub-corpus was an evaluative adjective “clear” (*n* = 157) which featured in 115/189 “successful” comments (dispersion – 60.8%) and had almost triple the number of hits than the second most frequent word “follow” (*n* = 53). Participants drew on this adjective to convey that features needed to be “clear” i.e. easy to find, appropriate, and comprehensible in terms of language (not uncommon acronym) and content. Common collocates of “clear” were “diagnosis” (Log-likelihood (LL) = 163) and “plan” (LL = 175.2). This highlights the importance of the “clearness” of these items to GPs which was exemplified in the results.

**Table 6:** Top 25 frequency results for sub-corpora comment content words

“Successful” letter comments sub-corpus				“Unsuccessful” letter comments sub-corpus			
Rank	No. of hits	Relative frequency (per 10,000 words)	Content word	Rank	No. of hits	Relative frequency (per 10,000 words)	Content word
1	157	750	clear	1	41	151	GP
2	53	253	follow	2	40	148	follow
3	50	239	plan	3	37	136	diagnosis
4	44	210	diagnosis	4	36	133	patient
5	38	182	good	5	32	118	discharge
6	38	182	summary	6	29	107	medication
7	35	167	discharge	7	26	96	summary
8	34	162	GP	8	23	85	clear
9	33	158	information	9	22	81	unclear
10	26	124	medication	10	21	77	information
11	21	100	detailed	11	20	74	poor
12	20	96	investigations	12	18	66	letter
13	20	96	medications	13	15	55	advice
14	19	91	treatment	14	14	52	hospital
15	18	86	given	15	13	48	handwritten
16	18	86	management	16	12	44	admission
17	17	81	action	17	12	44	dose
18	16	76	changes	18	10	37	medications
19	16	76	concise	19	10	37	plan
20	16	76	history	20	10	37	started
21	15	72	clearly	21	9	33	action
22	13	62	advice	22	9	33	blood
23	13	62	patient	23	9	33	details
24	12	20	admission	24	9	33	Indication
25	12	20	details	25	9	33	investigations

**Table 7:** Summary of GP comment analysis findings

Successful letter comments	Unsuccessful letter comments
*Discharging physician details* *•*Name of responsible consultant and discharging physician and their role *Clinical summary elements* *•*Diagnosis clear •Clear clinical summary •Clear results and interpretations of investigations/tests clearly recorded •Treatment given in hospital clear •Clear reason for admission •Clear history *Follow up or actions* *•*Clear follow up & management plan •Clear action plan to include appropriate actions for GP and why •Follow up arranged •If relevant, appointments organised •Clearly stated if no follow up or further action is required *Medication information* *•*Clear medication changes & why •Medication changes highlighted in GP action so not missed •Explicit if no medications changed •Advised medication monitoring and recommendations moving forward *Patient communication* *•*Information given to patient is clear *Letter style* *•*Information relevant and “concise” •Letter legible or readable *Other* •All information described as necessary included in letter •If relevant, home/social situation	*Discharging physician details* *•*No discharging physician name and position *Clinical summary elements* *•*No diagnosis or no clear diagnosis •No details of treatment (given and/or planned) •No indication of tests carried out or results •No information about reason for admission •Cause of admission not addressed *Follow up or actions* *•*No advice to GP for ongoing management •No or unclear follow up plan or arrangements •GP asked to make referrals hospital should have •Advice to GP described as vague and not helpful •Request for GP to chase results •Unrealistic GP blood test requests (<1 week) *Medication information* *•*No medication details •Not clear why medication changed •Says no GP action but changes made to medications •No medication dosing or duration •GP asked to prescribe specialist-only medication •Medication not dispensed *Patient communication* *•*Information given to patient not indicated or no information given to patient *Letter style* *•*Use of uncommon acronyms without explanation •Illegible/ letter is handwritten and difficult to read *Other* •No discharge date •No patient address •Incorrect information in summary •Key details omitted e.g. antibiotic given •Multiple addendums •Letter arrived late to GP/took a long time

Hospital survey respondents generally rated their letters highly; the median for all questions was either 7 or 8 (scale rating 1–9) (see additional file 4).

### Features of unsuccessful discharge letters

Only one summary had all 15 content components (0.3%). No single component was present in every summary despite the ubiquity of some of these components (e.g. reason for admission). The following components were infrequent (20–50%) within the sample: information given to patient (33.3%), reasons for medication changes (26.9%), explained medical jargon (23.5%). Two components were rarely present (< 20% of letters): explained acronyms (18.7%), patient concerns/wishes (8.3%). Unexplained acronyms were found in 81.3% of letters although differences between successful and unsuccessful letters were not significant (*X*^2^ = 3.482, *p* = 0.062). The feature of unexplained medical jargon was present in 76.5% of letters including 84.4% of unsuccessful letters (*X*^2^ = 7.095, *p* = 0.008).

For the 44 letters where both GP and HP gradings were available, 24 (54.5%) were rated as successful by the GPs and 36 (81.8%) were rated as successful by the HPs. There was overall agreement on letter successfulness in 24 cases (54.5%); this included 20 cases that were assessed as successful and four cases that were rated as unsuccessful. For 20 (45.5%) letters there was disagreement on the quality of the letter; this comprised 16 where the letter was graded successful by the hospital clinician but unsuccessful by the GP and the reverse occurred for 4 cases. These results were not statistically significant (k = 0.035, *p* = 0.775).

Question 15 in the hospital survey provided a free text space for respondents to comment further or provide reasons for their answers. Thirty-two respondents (69.6%) answered this question, answers generally consisted of 2–3 sentences ranging from 15 to 101 words. Several respondents identified barriers to providing successful discharge letters:

1. Juniors writing letters (*“These pro forma letters are often wrong, delegated to most junior doctors who may not even be on my team.”* Consultant)
2. Time restrictions (*“A good detailed discharge summary depends on the amount of time the doctor has to write it. At a busy*? *night shift, this is very difficult.”* Junior doctor) (*“Often we have short of doctors on the ward and we cannot spend too much time on discharge letters.”* Junior doctor)
3. Writing letters from patient notes and/or where patient not known to hospital clinician (*“We often have to retrospectively completed EDs [sic] after the patient is discharged - having only medical notes to go by - info can be limited.”* Nurse/ACP)
4. Issues with computer system/template (*“Our discharge letters are autogenerated from diagnostic and treatment PBR codes [sic]. We have no individual input into their quality/content”* Consultant) (*“There is a limit to “words“ what you can put [sic] on certain “text boxes“ hence sometimes whole information can’t be put on discharge summaries”* Consultant)
5. Lack of support and training (*“I feel perhaps further support from senior clinicians early on in the [TRAINING] will enable more concise discharge letters and enable [HOSPITAL ROLE] to have more confidence in completing them”* Nurse/ACP).

## Discussion

### Key findings

GP-graded “successful” letters generally adhered to national standards whereas “unsuccessful” letters did not. Successful letters more frequently included a range of elements, with statistically significant differences in the inclusion of details about reason for admission, diagnosis, medication changes, reasons for medication changes, hospital plan/actions, GP plan, information to patient, tests/procedures performed, and test/examination results. Hospital clinicians tended to rate their letters as being of greater quality than was evident from the assessment made by GPs. Hospital clinicians identified several barriers to producing “successful” letters, such as: juniors writing letters, time limitations, writing letters from patient notes retrospectively, and restrictions of electronic templates. Such barriers need addressing through increased training and organisational initiatives. Relatedly, the results demonstrated that the discharge letters within the sample were often incomplete.

Corpus linguistic analysis of the GP letter comments led to insights on GPs’ views on discharge letters (see Table 7 for summary). Importantly, “clear” was ranked top in the “successful” comment sub-corpus, which suggests that clarity may be one of the most important elements of a “successful” letter from a GP perspective. This highlights that it is important for discharge letters to be comprehensible, relevant, and concise. Letters that GPs rated “successful” tended to have particular content features: reason for admission, diagnosis, follow up and management plan, medication changes and reasons, GP actions, treatment, investigations and results, discharging physician details, and information provided to the patient. “Unsuccessful” letters (37.6% of sample) either omitted some of the above components or they were unclear, as detailed in Table 7. Moreover, GP comments revealed that “unsuccessful” letters may contain unexplained uncommon acronyms or jargon and/or actions GPs deemed inappropriate or unreasonable (e.g. requests for GPs to chase hospital results).

Although guidelines [37, 38, 57] recommend unexplained acronyms and jargon should be avoided or at least minimised, they were identified in the majority of the sample. This poses a substantial barrier in terms of letter accessibility for patients and GPs not familiar with the specialty; this is an important issue in light of current good practice which recommends copying letters to patients [36, 37].

### Strengths and limitations

The sample size was large enough to identify common drivers for GP assessed “successful” letters but small enough for qualitative analyses of all GP comments. By asking GPs to select letters that were no more than 3 weeks’ old at the time of selection, we aimed to improve participant recall and reduce biasing of the sample through GPs selecting particularly memorable (either markedly “successful” or “unsuccessful”) historical letters. Selecting “successful” and “unsuccessful” cases acted as a stimulus for participants to provide their views on how written discharge communications could be improved. An advantage of the sampling strategy was maximisation of variation. As planned, the letter sample exhibited diversity particularly in relation to reason for admission, and specialties. However, a limitation of this process was that it did not provide information on the average quality of National Health Service (NHS) discharge letters “typically” received in General Practice. Hence, it was not the aim of this study to describe what proportion of letters received by GPs in the setting studied are “successful” or “unsuccessful”.

Although the study was limited to a small geographical area, it is likely that the views about what constitutes a successful letter are widely generalizable. However, generalisability is limited by the exclusion criteria [27] i.e. certain categories of hospital discharge (e.g. related to children, mental health problems) were excluded. Research involving these excluded groups as well as a wider range of specialist settings is needed to understand discharge summary quality in other settings. Further research is also needed to determine if patient level characteristics such as polypharmacy, multimorbidity, age, and hospital length of stay influence the quality of discharge letters.

Content analysis was strengthened by the systematic approach; each document was subjected to the same categorisation criteria, and the criteria themselves were justifiable, grounded in national standards. Our content analysis was limited by the binary codes “successful” and “unsuccessful” which may have oversimplified complex or borderline cases and impacted statistical comparisons. Content coding was also categorical, depending on a feature being present in the letter or not. Furthermore, we were not able to link the discharge summaries to the wider medical record directly, the content coding therefore could not account for counterfactual information. For some features, absence of inclusion may reflect that the element of content (e.g. medication change or investigation) was not applicable to the care that the patient had received. However, without this being stated within the letter, this could not be determined. Generally, if the reason for absence was clear (e.g. letter stated that no medication changes were made), than this was coded as feature presence whereas if it was unclear or entirely missing from the letter than this was coded as feature absence. Additionally, the content analysis was not exhaustive of all aspects of discharge letters; outpatient clinics and emergency department discharge letters were excluded in order to reduce the effect of confounding factors. Future research should consider those categories and elements which have not been covered here. The effect (or not) of physician experience on letter quality warrants further investigation but would require a very large sample size in order to generate statistically significant findings about professional roles that rarely write discharge summaries.

The hospital clinician survey response rate of 14.9% was below the target of 50%. The survey target response rate was based on previous relevant survey studies [58, 59], although studies were found which had lower rates closer to our final figure: 19% [60], 13% [61]. Possible factors which reduced the hospital clinician response rate were: time constraints, survey advertisement strategy, junior doctor rotations, delays between writing letter and receiving survey.

GPs and hospital clinicians did not view each other’s letter gradings and comments. This was a strength in that hospital clinician recruitment and participation was not biased by the GP’s assessment. However, it did mean that feedback on the letter from the primary care perspective could not be provided to participating hospital clinicians on an individual basis; this could have been useful for professional development, and is a potential avenue for future work.

### Implications for practice

Missing information on discharge summaries could lead to errors in clinical care and might also affect patients’ understanding of what happened to them in hospital. The findings from this study indicate that important information is missing from many discharge summaries, including those graded “successful”. This is despite standards stipulating mandatory content of discharge summaries to include the US *Joint Commission* discharge summary standards [62, 63] and the UK Royal College of Physicians [35, 64] guidelines as well as the more recent PRSB eDischarge standard [40, 65] which allows Systematized Nomenclature of Medicine Clinical Terms (SNOMED) [66] coded information to be transferred directly between hospitals and GPs. Our findings suggest that failure for hospitals and clinicians to uniformly implement discharge letter guidance into practice is continuing to contribute to unsuccessful communication between hospitals and general practice; this has implications for continuity of care and patient safety. We make the following suggestions to increase uptake of guidelines into practice:

1. Promotion of this work and related studies to highlight the importance of guidelines for discharge summary creation and to build the evidence base behind guidelines
2. Involving patients in discharge communication

The elements of a “successful” letter identified in this study largely fit with PRSB discharge guidelines [40, 65] but also indicated scope for further development around some of the areas to include GP actions, treatment, and writing style. Delegation to GPs of following up on results from investigations undertaken while in hospital may create inter-professional tension [67, 68], but this practice continues despite national guidance to the contrary [69]. “Clear” was the number one content word within “successful” comments and so the importance of clarity and style to GPs is evident. GPs highlighted issues within the sample letters in regard to unexplained acronyms and uncommon jargon which can pose a clinical risk. This suggests a need for greater emphasis on the writing style within discharge summaries. Through identifying contributing factors towards “unsuccessful” letters, the research highlights a need for increased training and support for clinicians on letter writing as well as organisational initiatives such as evaluating implementation of guidelines [70] and ongoing audit to ensure that the quality of discharge letters minimises patient risk.

## Conclusions

This study examined the content and quality of discharge letters in respect of UK guidelines and standards, although the recommendations may have relevance to other healthcare systems. This study has highlighted barriers to producing high quality discharge summaries; these may be addressed through training and organisational initiatives. The sampled discharge summaries tended to lack components considered key by GPs, particularly information regarding what the patient has been told and GP actionable components. Poor discharge communication carries risks and represents a possible missed opportunity for effective communication and safe patient transition into the community setting. Letter quality hinged upon component inclusion and the clarity and relevance of the details to the specific case. In conclusion, this study outlines shortcomings of discharge summaries and ways in which discharge communication may be improved.

## Supplementary Information

**Additional file 1:.** GP letter selection template

**Additional file 2:.** Hospital clinician survey

**Additional file 3:.** Final content analysis categorisation coding system.

**Additional file 4:.** Summary of hospital clinician survey results.

## Data Availability

The majority of datasets supporting the conclusions of this article are included within the article (and its additional files). However, the full datasets generated during the current study are not publicly available due to the sensitive and identifiable nature of the data. Despite names and other identifiers being removed, the in-depth nature of the discharge letters may mean that patients could be identified. The anonymised survey results are found in additional file 4. Summaries of data characteristics have been provided for relevant data sets.

## Abbreviations

ACP: Advanced Clinical Practitioner
GP: General Practitioner (UK family or primary care physician)
LL: Log Likelihood
NHS: (UK) National Health Service
PRSB: The Professional Record Standards Body
SNOMED: Systematized Nomenclature of Medicine Clinical Terms
SPSS: Statistical Package for the Social Sciences
UK: United Kingdom
