# Supplementary material for: What makes a “successful” or “unsuccessful” discharge letter? Hospital clinician and General Practitioner assessments of the quality of discharge letters
Source: BMC Health Serv Res. 2021 Apr 15;21:349. doi: 10.1186/s12913-021-06345-z (PMC8048210; doi:10.1186/s12913-021-06345-z)
Supplement: Supplementary file 1 — Additional file 1:. GP letter selection template [file 12913_2021_6345_MOESM1_ESM.docx]

| No. of letters selected | Patient name (to be removed during redaction) | Patient Unique research ID (to be added during redaction) | Categorisation (**Unsuccessful** OR **successful** discharge letter example) | Reason for selection & categorisation (e.g. any key good or bad points about letter) |
| --- | --- | --- | --- | --- |
| *EXAMPLE*  *(Before redaction)*  *(after redaction)* | *Mr Joe Smith*  *………………..* | *P0001* | *Unsuccessful*  *Unsuccessful* | *Bad points:*  *Medication alterations poorly outlined and information given to patient not explained* |
| 1 |  |  |  |  |
| 2 |  |  |  |  |
| 3 |  |  |  |  |

*More rows to be added as needed…*
