# Supplementary material for: What makes a “successful” or “unsuccessful” discharge letter? Hospital clinician and General Practitioner assessments of the quality of discharge letters
Source: BMC Health Serv Res. 2021 Apr 15;21:349. doi: 10.1186/s12913-021-06345-z (PMC8048210; doi:10.1186/s12913-021-06345-z)
Supplement: Supplementary file 2 — Additional file 2:. Hospital clinician survey [file 12913_2021_6345_MOESM2_ESM.docx]

|  | Katharine WeetmanPhD StudentTel: 02476 151077 |
| --- | --- |
|  |  |

**Hospital Clinician Survey** Version 1.0 11.04.18

**Study Title: An Investigation of Written Discharge Communication between Hospital Clinicians, GPs and Patients**

We are interested in hearing your views about current practices of discharge communication, what you currently do and how you think the process could be improved.

GPs from across Coventry, Rugby, South & North Warwickshire and Herefordshire have selected a variety of discharge letters for this study and **you were the named writer of one of these letters.**

We have enclosed a redacted copy of this letter for your reference and reflection.

Please take a few minutes to fill out this survey on discharge communication for hospital clinicians. This survey is not anticipated to take up more than 5-15 minutes of your time.

**If you complete and return this survey you are consenting to take part** and for **your data to be used** for this discharge communication research study. If you would like further information, a hospital clinician (HP) participation information sheet is enclosed.

Electronic versions of the survey are available please email [K.Weetman@warwick.ac.uk](mailto:K.Weetman@warwick.ac.uk).

If you receive more than one survey invitation about different discharge letters/summaries you wrote or signed, please complete no more than 2 surveys in total.

**Research ID: HP [NUMBER]**

**Site (hospital):**

Please state your current professional grade/role _________________________________

Which specialty/ward were you working on when you wrote the attached letter? _____________________________________________________________________________

Please state the year you clinically qualified __________________________________________

Please state in which country you completed your medical training ____________________________

*Please note there is space at the end of this survey (Question 15) for you to comment and provide reasons for any of your answers.*

1. Please rate, in your opinion, the quality of the attached discharge letter you wrote:

Low Quality 1 2 3 4 5 6 7 8 9 High Quality

1. How does this letter quality compare to other letters you write?

better  worse  same

If answered ‘better’ or ‘worse’, please explain:

_______________________________________________________________________________

1. Please rate, in your opinion, how **clear** you think the attached discharge letter is to a GP:

Unclear 1 2 3 4 5 6 7 8 9 Very clear

1. Please rate how informative you think the **diagnosis** in the attached discharge letter is to a GP:

Uninformative 1 2 3 4 5 6 7 8 9 Informative

1. Please rate how informative you think the **medication plan** in the attached discharge letter is to a GP:

Uninformative 1 2 3 4 5 6 7 8 9 Informative

1. Please rate how informative you think the **care** **management plan (including any required follow up)** in the attached discharge letter is to a GP:

Uninformative 1 2 3 4 5 6 7 8 9 Informative

1. Please rate, in your opinion, how clear **(comprehensible)** you think the attached discharge letter is to a **PATIENT**:

Unclear 1 2 3 4 5 6 7 8 9 Very clear

1. How often do you copy patients into discharge letters?

Never  Occasionally  Most of the time  Always

1. How often do you think patients should be copied into/given/sent hospital discharge letters/summaries?

Never  Occasionally  Most of the time  Always

1. Do you feel all patients should be offered a choice of whether or not they receive/are given a hospital discharge letter?

Yes  No

1. Do you think patients should receive a direct copy of the discharge letter sent to the GP or a personalised patient discharge letter?

GP copy  Personalised letter  Neither  Both

1. What is your preferred discharge summary/letter form?

dictated/free text letters  structured letters  No preference  other _________________

1. Should patients receiving hospital discharge letters/summaries be an opt-in or opt-out system?

opt-in  opt-out  Neither  No preference

1. How often do you think your discharge letter writing is in line with the Department of Health “Copying letters to patients: good practice guidelines”?

Never  Occasionally  Most of the time  Always  Unfamiliar with guidelines

1. Please use this space to provide reasons for any of your answers or share any additional comments about discharge communication or how the process may be improved:

|  |
| --- |
|  |
|  |
|  |
|  |
|  |
|  |
|  |
|  |
|  |

**Thank you for taking the time to fill out our survey. We rely on your feedback to help us further communication research.**

Please return your survey by:

EMAIL: [K.Weetman@warwick.ac.uk](mailto:K.Weetman@warwick.ac.uk) OR POST TO:

**Katharine Weetman, Primary Care, Warwick Medical School, University of Warwick, Coventry, CV4 7AL**

Surveys may also be returned to your Research and Development department via internal post for transfer to the research team. The research team can also collect the survey(s), please email [K.Weetman@warwick.ac.uk](mailto:K.Weetman@warwick.ac.uk) to request this.

**If you are interested in hearing about the results of our research or further opportunities to get involved please complete the below (optional):**

A. I agree to my contact details being kept for the researcher to contact me for any further research activities for this project.

Yes  No

B. I would like the research team to share the research results with me.

Yes  No

If yes to A or B above, please provide your preferred contact details:

__________________________________________________________________________________________

At Warwick University, we strive for equal research opportunities for all. Where possible, we monitor patients and staff recruited for research to assess the sample diversity. If you are happy to provide any personal information about yourself, please complete below. **Please note: providing this information is not required for you to participate in this research.**

Hospital professional role:…...............

Age: ……………. Gender: …………………………………………….

Ethnicity: ………………………………………….

Religion: ……………………………………………
