# Supplementary material for: What makes a “successful” or “unsuccessful” discharge letter? Hospital clinician and General Practitioner assessments of the quality of discharge letters
Source: BMC Health Serv Res. 2021 Apr 15;21:349. doi: 10.1186/s12913-021-06345-z (PMC8048210; doi:10.1186/s12913-021-06345-z)
Supplement: Supplementary file 3 — Additional file 3:. Final content analysis categorisation coding system. [file 12913_2021_6345_MOESM3_ESM.docx]

***Final headings and coding categorisation system for discharge letter content analysis***

| **Content feature heading** | **Description*** | **Content feature**  **present/absent** | **Guideline/standard from which item was primarily extracted/based** |
| --- | --- | --- | --- |
| *Discharging physician* | Name and role of discharging physician/person completing and authorising discharge summary. | *Yes/no* | *Royal College of Physicians (2013) ^(1)^ “Standards for the clinical structure and content of patient records”*. |
| *Reason for admission/ contact purpose* | The health problems and issues experienced by patient resulting in their admission/attendance. Explanatory statement of the purpose of the contact. | *Yes/no* | *Royal College of Physicians (2013) ^(1)^ “Standards for the clinical structure and content of patient records”*. |
| *Diagnosis* | Description of problem in as much detail as possible at time of discharge to include confirmed, differential or working diagnoses. | *Yes/no* | *Royal College of Physicians (2013) ^(1)^ “Standards for the clinical structure and content of patient records”*. |
| *Procedures/investigations performed (if any)* | Any procedures or investigations performed. This could include blood tests, imaging etc. | *Yes/no* | *Royal College of Physicians (2013) ^(1)^ “Standards for the clinical structure and content of patient records”*. |
| *Procedure/investigation results and examination findings* | Results of investigations or tests including result value, interpretation and any plans for acting upon results. Record of any results of observations or examinations carried out (e.g. blood pressure, examination of skin). | *Yes/no* | *Royal College of Physicians (2013) ^(1)^ “Standards for the clinical structure and content of patient records”*. |
| *Medication name(s)* | Generic and/or brand name. | *Yes/no* | *Royal College of Physicians (2013) ^(1)^ “Standards for the clinical structure and content of patient records”*. |
| *Medication dose and frequency* | Amount of ingredients to be taken and frequency of administration. | *Yes/no* | *Royal College of Physicians (2013) ^(1)^ “Standards for the clinical structure and content of patient records”*. |
| *Medication changes* | Note when a drug has been stopped and another started. Highlight any new medications. Must explicitly state that there has been a change. | *Yes/no* | *Royal College of Physicians (2013) ^(1)^ “Standards for the clinical structure and content of patient records”*. |
| *Reasons for medication changes* | Explain reasons for any medication changes (e.g. patient intolerant) or new medications started. Must explicitly state why in terms of reason for medication change. | *Yes/no* | *Royal College of Physicians (2013) ^(1)^ “Standards for the clinical structure and content of patient records”*. |
| *Hospital plan/actions (Investigations/procedures requested or where results are pending)* | This should include name of investigation/appointment/test/procedure requested or details of pending communications, results or referrals to be actioned by the hospital. | *Yes/no* | *Royal College of Physicians (2013) ^(1)^ “Standards for the clinical structure and content of patient records”*. |
| *Patient’s and carer’s concerns, expectations and wishes* | Any concerns wishes or goals of patient or relevant carer/guardian/representative. | *Yes/no* | *Royal College of Physicians (2013) ^(1)^ “Standards for the clinical structure and content of patient records”*. |
| *Information and advice given to patient* | This should include what information was given and to whom. This can include oral and written information and may include cases where this is implicitly communicated. | *Yes/no* | *Royal College of Physicians (2013) ^(1)^ “Standards for the clinical structure and content of patient records”*. |
| *GP Plan, follow up and actions* | GP actions and follow up plan to include items which need repeating or undertaking in the future (e.g. repeat blood tests) and may include a recommended management plan or recommendations regarding medications. | *Yes/no* | *Royal College of Physicians (2013) ^(1)^ “Standards for the clinical structure and content of patient records”*. |
| *Acronyms* | Unexplained acronyms and abbreviations should be avoided with the exception of very commonplace abbreviations e.g. A&E. | *Yes/no* | *Department of Health (2003) ^(2)^ “Copying letters to patients: good practice guidelines”* |
| *Jargon* | Avoid unexplained jargon/medical terms. Lay terms should be used following medical terms. | *Yes/no* | *Department of Health (2003) ^(2)^ “Copying letters to patients: good practice guidelines”* |

* NB: description is sometimes direct wording extracted from document

**References**

1.Royal College of Physicians. Standards for the clinical structure and content of patient records. 2013 <https://www.rcplondon.ac.uk/projects/outputs/standards-clinical-structure-and-content-patient-records>.

2.*Department of Health*. Copying letters to patients: good practice guidelines [Report]. 2003. Available from: <https://webarchive.nationalarchives.gov.uk/20120504030618/http://www.dh.gov.uk/prod_consum_dh/groups/dh_digitalassets/@dh/@en/documents/digitalasset/dh_4086054.pdf>.
