# Supplementary material for: What makes a “successful” or “unsuccessful” discharge letter? Hospital clinician and General Practitioner assessments of the quality of discharge letters
Source: BMC Health Serv Res. 2021 Apr 15;21:349. doi: 10.1186/s12913-021-06345-z (PMC8048210; doi:10.1186/s12913-021-06345-z)
Supplement: Supplementary file 4 — Additional file 4:. Summary of hospital clinician survey results. [file 12913_2021_6345_MOESM4_ESM.docx]

***Hospital professional survey results***

*Q1 Please rate, in your opinion, the quality of the attached discharge letter you wrote (9pt scale)*

| **Descriptive Statistics** | | | | | | | | | |
| --- | --- | --- | --- | --- | --- | --- | --- | --- | --- |
|  | N | Minimum | Maximum | Mean | Std. Deviation | Skewness | | Kurtosis | |
|  | Statistic | Statistic | Statistic | Statistic | Statistic | Statistic | Std. Error | Statistic | Std. Error |
| Q1 (Quality of letter) | **44** | **1** | **9** | **6.61** | **2.244** | **-1.522** | **.357** | **1.725** | **.702** |
| Valid N (listwise) | **44** |  |  |  |  |  |  |  |  |

| **Q1 (Quality of letter)** | | | | | |
| --- | --- | --- | --- | --- | --- |
|  | | Frequency | Percent | Valid Percent | Cumulative Percent |
| Valid | 1 | **4** | **8.7** | **9.1** | **9.1** |
|  | 2 | **1** | **2.2** | **2.3** | **11.4** |
|  | 5 | **3** | **6.5** | **6.8** | **18.2** |
|  | 6 | **6** | **13.0** | **13.6** | **31.8** |
|  | 7 | **12** | **26.1** | **27.3** | **59.1** |
|  | 8 | **12** | **26.1** | **27.3** | **86.4** |
|  | 9 | **6** | **13.0** | **13.6** | **100.0** |
|  | Total | **44** | **95.7** | **100.0** |  |
| Missing | System | **2** | **4.3** |  |  |
| Total | | **46** | **100.0** |  |  |

*Q2 How does this letter quality compare to other letters you write?*

| **Q2 (compared with other letters)** | | | | | |
| --- | --- | --- | --- | --- | --- |
|  | | Frequency | Percent | Valid Percent | Cumulative Percent |
| Valid | better | **2** | **4.3** | **4.3** | **4.3** |
|  | same | **34** | **73.9** | **73.9** | **78.3** |
|  | worse | **10** | **21.7** | **21.7** | **100.0** |
|  | Total | **46** | **100.0** | **100.0** |  |

*Q3 Please rate, in your opinion, how* ***clear*** *you think the attached discharge letter is to a GP (9pt scale)*

| **Descriptive Statistics** | | | | | | | | | |
| --- | --- | --- | --- | --- | --- | --- | --- | --- | --- |
|  | N | Minimum | Maximum | Mean | Std. Deviation | Skewness | | Kurtosis | |
|  | Statistic | Statistic | Statistic | Statistic | Statistic | Statistic | Std. Error | Statistic | Std. Error |
| Q3 (GP clearness rating) | **45** | **1** | **9** | **7.62** | **1.709** | **-2.232** | **.354** | **5.678** | **.695** |
| Valid N (listwise) | **45** |  |  |  |  |  |  |  |  |

| **Q3 (GP clearness rating)** | | | | | |
| --- | --- | --- | --- | --- | --- |
|  | | Frequency | Percent | Valid Percent | Cumulative Percent |
| Valid | 1 | **1** | **2.2** | **2.2** | **2.2** |
|  | 3 | **2** | **4.3** | **4.4** | **6.7** |
|  | 6 | **4** | **8.7** | **8.9** | **15.6** |
|  | 7 | **6** | **13.0** | **13.3** | **28.9** |
|  | 8 | **18** | **39.1** | **40.0** | **68.9** |
|  | 9 | **14** | **30.4** | **31.1** | **100.0** |
|  | Total | **45** | **97.8** | **100.0** |  |
| Missing | System | **1** | **2.2** |  |  |
| Total | | **46** | **100.0** |  |  |

*Q4 Please rate how informative you think the* ***diagnosis*** *in the attached discharge letter is to a GP (9pt scale)*

| **Descriptive Statistics** | | | | | | | | | |
| --- | --- | --- | --- | --- | --- | --- | --- | --- | --- |
|  | N | Minimum | Maximum | Mean | Std. Deviation | Skewness | | Kurtosis | |
|  | Statistic | Statistic | Statistic | Statistic | Statistic | Statistic | Std. Error | Statistic | Std. Error |
| Q4 (GP diagnosis information) | **46** | **1** | **9** | **7.17** | **2.434** | **-1.662** | **.350** | **1.651** | **.688** |
| Valid N (listwise) | **46** |  |  |  |  |  |  |  |  |

| **Q4 (GP diagnosis information)** | | | | | |
| --- | --- | --- | --- | --- | --- |
|  | | Frequency | Percent | Valid Percent | Cumulative Percent |
| Valid | 1 | **3** | **6.5** | **6.5** | **6.5** |
|  | 2 | **3** | **6.5** | **6.5** | **13.0** |
|  | 5 | **1** | **2.2** | **2.2** | **15.2** |
|  | 6 | **3** | **6.5** | **6.5** | **21.7** |
|  | 7 | **6** | **13.0** | **13.0** | **34.8** |
|  | 8 | **14** | **30.4** | **30.4** | **65.2** |
|  | 9 | **16** | **34.8** | **34.8** | **100.0** |
|  | Total | **46** | **100.0** | **100.0** |  |

*Q5 Please rate how informative you think the medication plan in the attached discharge letter is to a GP (9pt scale)*

| **Descriptive Statistics** | | | | | | | | | |
| --- | --- | --- | --- | --- | --- | --- | --- | --- | --- |
|  | N | Minimum | Maximum | Mean | Std. Deviation | Skewness | | Kurtosis | |
|  | Statistic | Statistic | Statistic | Statistic | Statistic | Statistic | Std. Error | Statistic | Std. Error |
| Q5 (GP medication plan info.) | **42** | **1** | **9** | **7.52** | **2.027** | **-1.964** | **.365** | **3.834** | **.717** |
| Valid N (listwise) | **42** |  |  |  |  |  |  |  |  |

| **Q5 (GP medication plan info.)** | | | | | |
| --- | --- | --- | --- | --- | --- |
|  | | Frequency | Percent | Valid Percent | Cumulative Percent |
| Valid | 1 | **2** | **4.3** | **4.8** | **4.8** |
|  | 3 | **1** | **2.2** | **2.4** | **7.1** |
|  | 5 | **2** | **4.3** | **4.8** | **11.9** |
|  | 6 | **4** | **8.7** | **9.5** | **21.4** |
|  | 7 | **4** | **8.7** | **9.5** | **31.0** |
|  | 8 | **12** | **26.1** | **28.6** | **59.5** |
|  | 9 | **17** | **37.0** | **40.5** | **100.0** |
|  | Total | **42** | **91.3** | **100.0** |  |
| Missing | System | **4** | **8.7** |  |  |
| Total | | **46** | **100.0** |  |  |

*Q6 Please rate how informative you think the care management plan (including any required follow up) in the attached discharge letter is to a GP (9pt scale)*

| **Descriptive Statistics** | | | | | | | | | |
| --- | --- | --- | --- | --- | --- | --- | --- | --- | --- |
|  | N | Minimum | Maximum | Mean | Std. Deviation | Skewness | | Kurtosis | |
|  | Statistic | Statistic | Statistic | Statistic | Statistic | Statistic | Std. Error | Statistic | Std. Error |
| Q6 (GP care management plan info.) | **45** | **1** | **9** | **7.40** | **1.935** | **-1.820** | **.354** | **3.714** | **.695** |
| Valid N (listwise) | **45** |  |  |  |  |  |  |  |  |

| **Q6 (GP care management plan info.)** | | | | | |
| --- | --- | --- | --- | --- | --- |
|  | | Frequency | Percent | Valid Percent | Cumulative Percent |
| Valid | 1 | **2** | **4.3** | **4.4** | **4.4** |
|  | 4 | **1** | **2.2** | **2.2** | **6.7** |
|  | 5 | **4** | **8.7** | **8.9** | **15.6** |
|  | 6 | **1** | **2.2** | **2.2** | **17.8** |
|  | 7 | **11** | **23.9** | **24.4** | **42.2** |
|  | 8 | **10** | **21.7** | **22.2** | **64.4** |
|  | 9 | **16** | **34.8** | **35.6** | **100.0** |
|  | Total | **45** | **97.8** | **100.0** |  |
| Missing | System | **1** | **2.2** |  |  |
| Total | | **46** | **100.0** |  |  |

*Q7 Please rate, in your opinion, how clear (comprehensible) you think the attached discharge letter is to a PATIENT (9pt scale)*

| **Descriptive Statistics** | | | | | | | | | |
| --- | --- | --- | --- | --- | --- | --- | --- | --- | --- |
|  | N | Minimum | Maximum | Mean | Std. Deviation | Skewness | | Kurtosis | |
|  | Statistic | Statistic | Statistic | Statistic | Statistic | Statistic | Std. Error | Statistic | Std. Error |
| Q7 (PATIENT comprehensible) | **45** | **1** | **9** | **6.20** | **2.312** | **-.798** | **.354** | **-.375** | **.695** |
| Valid N (listwise) | **45** |  |  |  |  |  |  |  |  |

| **Q7 (PATIENT comprehensible)** | | | | | |
| --- | --- | --- | --- | --- | --- |
|  | | Frequency | Percent | Valid Percent | Cumulative Percent |
| Valid | 1 | **2** | **4.3** | **4.4** | **4.4** |
|  | 2 | **2** | **4.3** | **4.4** | **8.9** |
|  | 3 | **5** | **10.9** | **11.1** | **20.0** |
|  | 4 | **1** | **2.2** | **2.2** | **22.2** |
|  | 5 | **3** | **6.5** | **6.7** | **28.9** |
|  | 6 | **7** | **15.2** | **15.6** | **44.4** |
|  | 7 | **9** | **19.6** | **20.0** | **64.4** |
|  | 8 | **10** | **21.7** | **22.2** | **86.7** |
|  | 9 | **6** | **13.0** | **13.3** | **100.0** |
|  | Total | **45** | **97.8** | **100.0** |  |
| Missing | System | **1** | **2.2** |  |  |
| Total | | **46** | **100.0** |  |  |

*Q8 How often do you copy patients into discharge letters?*

| **Q8 (how often do you copy patients into discharge letters)** | | | | | |
| --- | --- | --- | --- | --- | --- |
|  | | Frequency | Percent | Valid Percent | Cumulative Percent |
| Valid | always | **17** | **37.0** | **37.0** | **37.0** |
|  | mostly | **8** | **17.4** | **17.4** | **54.3** |
|  | never | **10** | **21.7** | **21.7** | **76.1** |
|  | occasionally | **11** | **23.9** | **23.9** | **100.0** |
|  | Total | **46** | **100.0** | **100.0** |  |

*Q9 How often do you think patients should be copied into/given/sent/hospital discharge letters/summaries?*

| **Q9 (how often do you think patients should be copied in)** | | | | | |
| --- | --- | --- | --- | --- | --- |
|  | | Frequency | Percent | Valid Percent | Cumulative Percent |
| Valid | always | **26** | **56.5** | **56.5** | **56.5** |
|  | mostly | **8** | **17.4** | **17.4** | **73.9** |
|  | never | **1** | **2.2** | **2.2** | **76.1** |
|  | occasionally | **11** | **23.9** | **23.9** | **100.0** |
|  | Total | **46** | **100.0** | **100.0** |  |

*Q10 Do you feel all patients should be offered a choice of whether or not they receive/are given a hospital discharge letter?*

| **Q10 (should all patients be offered choice)** | | | | | |
| --- | --- | --- | --- | --- | --- |
|  | | Frequency | Percent | Valid Percent | Cumulative Percent |
| Valid | no | **16** | **34.8** | **34.8** | **34.8** |
|  | yes | **30** | **65.2** | **65.2** | **100.0** |
|  | Total | **46** | **100.0** | **100.0** |  |

*Q11 Do you think patients should receive a direct copy of the discharge letter sent to the GP or a personalised patient discharge letter?*

| **Q11 (should patients receive direct or personalised)** | | | | | |
| --- | --- | --- | --- | --- | --- |
|  | | Frequency | Percent | Valid Percent | Cumulative Percent |
| Valid | both | **6** | **13.0** | **13.0** | **13.0** |
|  | GP copy | **23** | **50.0** | **50.0** | **63.0** |
|  | neither | **2** | **4.3** | **4.3** | **67.4** |
|  | personal | **15** | **32.6** | **32.6** | **100.0** |
|  | Total | **46** | **100.0** | **100.0** |  |

*Q12 What is your preferred discharge summary/letter form?*

| **Q12 (preferred letter form)** | | | | | | |
| --- | --- | --- | --- | --- | --- | --- |
|  | | Frequency | Percent | Valid Percent | Cumulative Percent |  |
| Valid | both | **1** | **2.2** | **2.2** | **2.2** |  |
|  | dictated | **9** | **19.6** | **19.6** | **21.7** |  |
|  | no preference | **6** | **13.0** | **13.0** | **34.8** |  |
|  | other | **2** | **4.3** | **4.3** | **39.1** |  |
|  | structured | **28** | **60.9** | **60.9** | **100.0** |  |
|  | Total | **46** | **100.0** | **100.0** |  |  |

*Q13 Should patients receiving hospital discharge letters/summaries be an opt-in or opt-out system?*

| **Q13 (system should be opt in or opt out)** | | | | | | |
| --- | --- | --- | --- | --- | --- | --- |
|  | | Frequency | Percent | Valid Percent | Cumulative Percent |  |
| Valid | neither | **6** | **13.0** | **13.0** | **13.0** |  |
|  | no preference | **12** | **26.1** | **26.1** | **39.1** |  |
|  | opt in | **10** | **21.7** | **21.7** | **60.9** |  |
|  | opt out | **18** | **39.1** | **39.1** | **100.0** |  |
|  | Total | **46** | **100.0** | **100.0** |  |  |

*Q14 How often do you think your discharge letter writing is in line with the Department of Health “Copying letters to patients: good practice guidelines”?*

| **Q14 (how often is your letter writing in line with guidelines)** | | | | | | |
| --- | --- | --- | --- | --- | --- | --- |
|  | | Frequency | Percent | Valid Percent | Cumulative Percent |  |
| Valid | always | **3** | **6.5** | **6.5** | **6.5** |  |
|  | mostly | **9** | **19.6** | **19.6** | **26.1** |  |
|  | never | **1** | **2.2** | **2.2** | **28.3** |  |
|  | occasionally | **7** | **15.2** | **15.2** | **43.5** |  |
|  | Unfamiliar with guidelines | **26** | **56.5** | **56.5** | **100.0** |  |
|  | Total | **46** | **100.0** | **100.0** |  |  |

*Q15 Please use this space to provide reasons for any of your answers or share any additional comments about discharge communication or how the process may be improved*

**Results not provided as free text answers may identify participants**

***Other outputs of results***

*Descriptive results (range and IQR) for Q1, 3-7*

|  | | **Q1** | **Q3** | **Q4** | **Q5** | **Q6** | **Q7** |
| --- | --- | --- | --- | --- | --- | --- | --- |
| N | Valid | 44 | 45 | 46 | 42 | 45 | 45 |
|  | Missing | 2 | 1 | 0 | 4 | 1 | 1 |
| Median | | 7.00 | 8.00 | 8.00 | 8.00 | 8.00 | 7.00 |
| Range | | 8 | 8 | 8 | 8 | 8 | 8 |
| Percentiles | 25 | 6.00 | 7.00 | 7.00 | 7.00 | 7.00 | 5.00 |
|  | 50 | 7.00 | 8.00 | 8.00 | 8.00 | 8.00 | 7.00 |
|  | 75 | 8.00 | 9.00 | 9.00 | 9.00 | 9.00 | 8.00 |

*Agreement between GP and HPs (Q1)* *HP grade coded unsuccessful 1-5 successful 6-9*

|  | | | | | **Q1 (Quality of letter)** | | | **Total** |
| --- | --- | --- | --- | --- | --- | --- | --- | --- |
|  |  |  |  |  | **successful** | **unsuccessful** | |  |
| GP letter grading | successful | | Count | | 20 | 4 | | 24 |
|  | unsuccessful | | Count | | 16 | 4 | | 20 |
| Total | | | Count | | 36 | 8 | | 44 |
| **Symmetric Measures** | | | | | | | | |
|  | | | **Value** | **Asymptotic Standard Error** | **Approximate T** | | **Approximate Significance** | |
| Measure of Agreement | | Kappa | .035 | .124 | .285 | | .775 | |
| N of Valid Cases | | | 44 |  |  | |  | |
